# Elucidating Methods for Isolation and Quantification of Exosomes: A Review

**DOI:** 10.1007/s12033-021-00300-3

**Published:** 2021-01-25

**Authors:** Talitha Keren Kurian, Soumyabrata Banik, Dharshini Gopal, Shweta Chakrabarti, Nirmal Mazumder

**Affiliations:** 1 Department of Biophysics, Manipal School of Life Sciences, Manipal Academy of Higher Education (MAHE), Manipal, Karnataka 576104 India; 2 Department of Bioinformatics, Manipal School of Life Sciences, Manipal Academy of Higher Education (MAHE), Manipal, Karnataka 576104 India

**Keywords:** Exosomes, Biomarkers, Therapeutics, Diagnosis

## Abstract

Exosomes are the smallest extracellular vesicles present in most of the biological fluids. They are found to play an important role in cell signaling, immune response, tumor metastasis, etc. Studies have shown that these vesicles also have diagnostic and therapeutic roles for which their accurate detection and quantification is essential. Due to the complexity in size and structure of exosomes, even the gold standard methods face challenges. This comprehensive review discusses the various standard methods such as ultracentrifugation, ultrafiltration, size-exclusion chromatography, precipitation, immunoaffinity, and microfluidic technologies for the isolation of exosomes. The principle of isolation of each method is described, as well as their specific advantages and disadvantages. Quantification of exosomes by nanoparticle tracking analysis, flow cytometry, tunable resistive pulse sensing, electron microscopy, dynamic light scattering, and microfluidic devices are also described, along with the applications of exosomes in various biomedical domains.

## Introduction

**Fig. 1:**
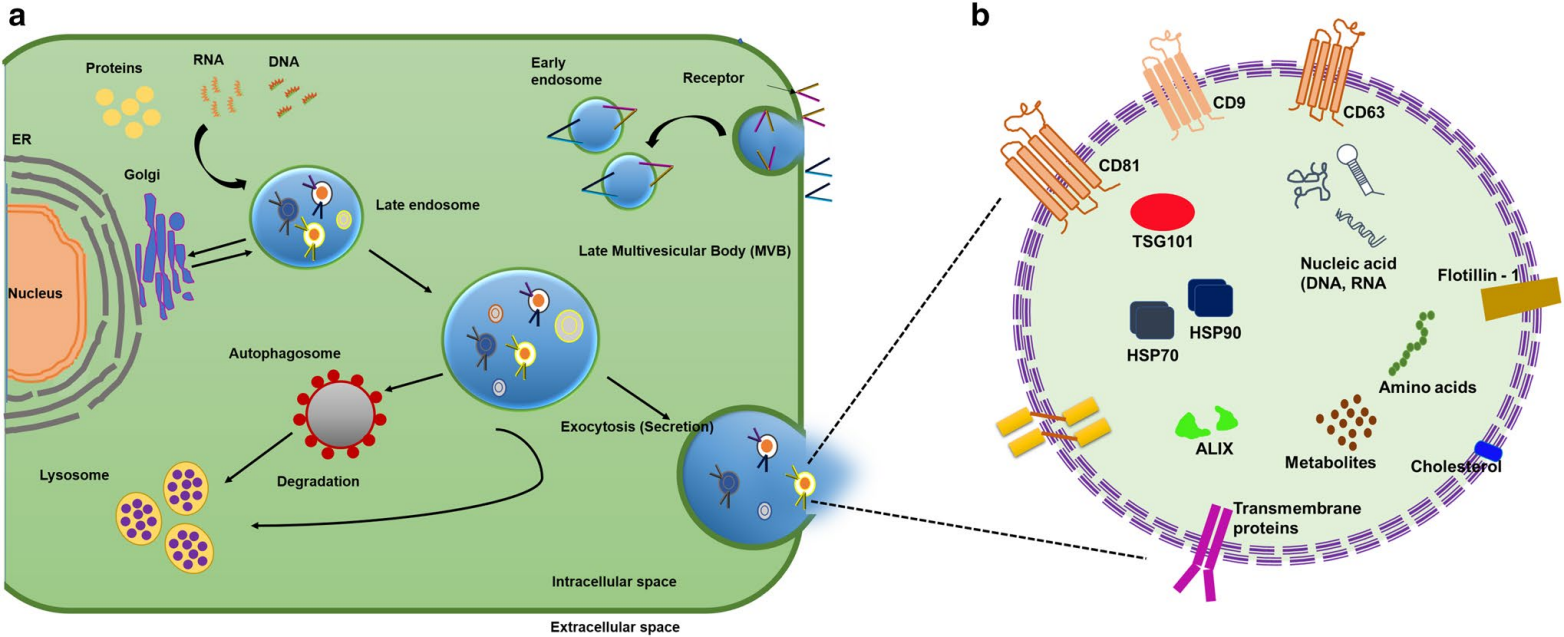
**a** Illustrates the process by which exosomes are produced and released from the cell; **b** shows the overall composition including the surface markers of an exosome released from the cell. Figure is reproduced from [8, 9] with permission from MDPI publisher.

Extracellular vesicles (EVs) are membrane-bound lipid vesicles. They are spherical secretory vesicles with a proteolipid bilayer membrane, their diameters ranging from 30–2000 nm [1]. Most eukaryotic cell types release these EVs as a means of intracellular communication and cellular waste removal. Apart from the proteins expressed on their membranes, EVs also contain other proteins and lipids, as well as genetic information (DNA, mRNAs, miRNAs, etc.). Based on the differences in their biogenesis, compositions, and functions, EVs are classified into microvesicles, exosomes and apoptotic bodies [2–4]. Figure 1a illustrates the process by which exosomes are produced and released from the cell, also the overall composition including the surface markers of an exosome has been shown in Fig. 1b. Further, the types of EVs and their various compositions have been summarized in Table 1 and 2. Micro vesicles (MVs) bud from the plasma membrane and are of 100 – 1000 nm in size. Due to the differences in their origins, proteins such as integrins, arrestin containing protein 1 (AARDC1) and P-selectin glycoprotein Ib (GPIb) are enriched in MVs [5]. They tend to contain more proteins that have undergone posttranslational modifications, including glycoproteins and phosphoproteins, compared to exosomes [6]. Dying cells that undergo apoptosis release apoptotic bodies from the plasma membrane, with diameters of 50 – 2000 nm. These vesicles contain DNA-binding histones and lack glycoproteins, directly opposite to exosomes [7].

**Table 1:** Comparison between extracellular vesicles

Types	Exosomes	Microvesicles	Apoptotic bodies	References
Origin	Endocytic pathway	Plasma membrane	Plasma membrane	[1, 9]
Size	30–150 nm	50–1000 nm	500–2000 nm	
Function	Intercellular communication	Intercellular communication	Facilitate phagocytosis	
Markers	Alix, Tsg101, tetraspanins (CD81, CD63, CD9), flotillin	Integrins, selectins, CD40	Annexin V, phosphatidylserine	
Contents	Proteins and nucleic acids (mRNA, miRNA, and other non-coding RNAs)	Proteins and nucleic acids (mRNA, miRNA, and other non-coding RNAs)	Nuclear fractions, cell organelles

**Table 2:** Different types/classes of protein/RNA/lipid content in EVs

Types/classes	Protein/lipid/RNA	References
MHC class II‐associated proteins	Protein	[10]
Tetraspanin proteins	Protein	[11]
HLA-DM	Protein	
Lamp-1, and Lamp-2	Protein	
GM1	Protein	[12]
Lyn, flotillin-1	Protein	
Stomatin	Protein	
Adaptor protein CD2AP	Protein	[13]
L-type lectin LMAN2	Protein	
Activating transcription factor 3 (ATF3)	Protein	[14]
Wilms tumor 1 (WT-1)	Protein	
Sphingomyelins	Lipid	[15]
Cardiolipins	Lipid	
Latent membrane protein 1 (LMP1)	Protein	[16]
Immunomodulatory protein galectin 9	Protein	
Heat shock protein (Hsc73)	Protein	[17]
Ubiquitinated proteins	Protein	[18]
Inhibitory protein CD59	Protein	[19]
Hexosylceramides	Lipid	[20]
Phosphatidylserine	Lipid	
Saturated fatty acids	Lipid	
mRNA, miRNA, lncRNA	RNA	
Heat shock protein (HSP70, HSP84)	Protein	[7, 21]
HLA-G1 proteins	Protein	[22]
SNARE synaptobrevin 2 (VAMP2)	Protein	[23]
SNX25, BTG1, PEDF, thrombospondin 2	Protein	[24]

Exosomes, often present in bodily fluids, are about 30–150 nm in size and are known to be the smallest type of extracellular vesicles. Previously thought of as cellular waste disposal systems, exosomes are now known for their functions in cell and signaling, antigen presentation, immune response modulation, and more. They play a role in tumor metastasis, with cancer cell-derived exosomes carrying information responsible for angiogenesis and cell proliferation [1]. They also serve a therapeutic purpose, as their biocompatibility, low immunogenicity, and ability to cross the blood–brain barrier, allowing them to be exploited for targeted drug delivery therapy [2]. Exosomes isolated from urine can also serve as biomarkers of genitourinary disorders and prostate cancer, while those extracted from the cerebrospinal fluid can be used to diagnose prion diseases and a few neurodegenerative disorders [3]. This role as biomarkers for disease detection and prognosis makes exosomes an ideal candidate for use in liquid biopsies, in which exosomes released by parent cells into circulating fluids (blood, urine) can be analyzed to identify the presence or severity of diseases. However, research into the role of exosomes as biomarkers for disease requires accurate measurement of their concentrations in clinical samples. This remains difficult due to the complexity of isolating pure exosomes from biological and cell culture fluids, and of detecting and enumerating them [25].

With the increasing interest in the field of exosomes, a comprehensive article highlighting the different methods for their isolation and detection is important. It will provide researchers with a broader understanding of each technique and allow them to choose the one most appropriate for their work. In this review, we focus on the methods for isolation and quantification of exosomes. First, a brief overview on the biogenesis and structures of EVs and exosomes is provided. We then discuss the different methods employed for exosome isolation and purification, and the advantages and disadvantages of each method. We also highlight the different applications of exosomes as biomarkers for diagnosis of various medical conditions and their potential therapeutic role.

## Exosome

### Discovery

In 1983, two papers independent of each other, by Harding et al. and Pan et al. described exosomes for the first time, only a week apart from each other. In these papers, the movement of labeled transferrin receptors (TfRs) from the plasma membrane into maturing reticulocytes was tracked. It was discovered that the transferrin receptors are taken into the cells, and then repackaged into small (~ 50 nm) vesicles inside them [8, 26]. These vesicles originally thought destined to be trafficked to lysosomes for destruction, are instead secreted out of the maturing blood reticulocytes into the extracellular space and were later coined “exosomes” [27, 28].

### Biogenesis and Release

Exosomes are formed as a product of the endosomal pathway unlike MVs and apoptotic bodies which also arise from the plasma membrane budding. As depicted in Fig. 1, the releasing of the exosomes is by the inward budding by forming endocytic vesicles. These vesicles will have an inside-out cell membrane externally and extracellular fluid internally [8]. Several endocytic vesicles will fuse to form an endosome, which later matures into multivesicular endosomes. During this, the outer membrane of the endosomes invaginates to generate small vesicles measuring about 50–90 nm in diameter, called intraluminal vesicles (ILVs) containing cytosolic content. These multivesicular endosomes or bodies (MVBs) have two fates: either as intermediates in intracellular protein degradation or in exosome formation. In the endolysosomal degradative pathway, proteins from the cell membrane or Trans-Golgi packaged into ILVs fuse with lysosomes which release enzymes to destroy proteins. Alternatively, these ILVs are routed to the cell surface, to deliver its material into the extracellular space as exosomes [29].

For the formation of MVBs, the sorting and packaging of their protein cargo depends on the endosomal sorting complex required for transport (ESCRT) such as ESCRT-0, I, II, III and associated proteins such as Vps4, Tsg101 and Alix. The ESCRT-0 complex interacts with the -I and -II complexes, once it is ubiquinated, which cause membrane deformation and form the neck of the budding membrane. Together, the complexes combine with ESCRT-III, which interacts with Vps4 to cleave the necks of the buds to form ILVs. However, recent evidence suggests that there could be an ESCRT-independent pathway for exosome formation and cargo loading, which uses raft-based lipid microdomains of the plasma membrane and associated proteins like tetraspanins [30]. The lipid rafts are enriched with sphingomyelinases, which convert sphingomyelin to ceramide, a sphingolipid that induces the spontaneous budding of the membrane to form ILVs [31]. The role of tetraspanins in vesicle formation is still being studied but is thought to induce negative curving of the membrane or vesicle scission. Tetraspanin-enriched microdomains (TEMs) are involved with cargo loading, sorting target receptors, signaling proteins, and cellular components into exosomes [32].

### Structure and Composition

Due to their small size, exosomes are not visible to the naked eye or under light microscopes and can only be visualized under an electron microscope. They appear as flattened spheres, most likely due to the preparation process for electron microscopy, which involves extreme dehydration resulting in the exosomes collapsing [9, 33]. In recent years, structural studies of exosomes have revealed that they contain certain lipids that help to maintain their biological activity, many of which are recorded in the exosome database ExoCarta. Exosome membranes are composed of a lipid bilayer, containing other biomolecules such as unsaturated lipids, cholesterol, phosphatidylserine, sphingomyelin, and gangliosides [34]. The high levels of unsaturated lipids and sphingomyelins in the exosome membrane may be the cause of its strength and rigidity, making it less susceptible to degradation outside the cell and more stable as a carrier [35]. However, the exosome lipid profile does not fully reflect that of their parent cells. They have minor differences compared to the membranes of their parent cells, namely containing increased proportions of cholesterol, sphingomyelin and phosphatidylserine, and less phosphatidylcholine and lyso(bis)phosphatidic acid [36]. The protein composition of extracellular vesicles is a good indicator of the subtype of EV, the mode of biogenesis and release, and the original cell type. However, regardless of these factors, all exosomes contain some common proteins, for instance, heat shock protein 84 (Hsp84), tumor susceptibility gene 101 (TSG101) and Alix, for transport mechanism [7]. The endosomal ESCRT is a collection of proteins required for the membrane infoldings to form MVBs [37]. Five protein complexes comprise the ESCRT machinery, ESCRT-0, ESCRT-I, ESCRT-II, ESCRT-III and Vps4-Vta1, and a few ESCRT-associated proteins. Among them, the soluble complexes are ESCRT-0, ESCRT-I, ESCRT-II and Vps4-Vta1 [23]. ESCRT complex components, GTPases, Rab, tetraspanin, and other proteins play a role in the recognition, uptake, release, and exosome internalization. Alix, TSG101, Hsp70, and CD9 were the exosome markers used in the Western blotting and electron microscopy techniques [21]. Other commonly found exosomal proteins include membrane adhesion proteins such as integrins, membrane transport/trafficking which include annexins and rab protein family, cytoskeletal components such as ezrin, actin, tubulin, cytokeratins and myosin), and lysosome membrane protein 2, cathepsin D, CD63, LAMP-1/2 which are the lysosomal markers, antigen presenting proteins such as HLA class I and II/peptide complexes, metabolic enzymes such as GAPDH and pyruvate, heat shock proteins, proteases (ADAM10, DPEP1, ST14), kinases, transporters (ATP7A, ATP7B, MRP2, SLC1A4, SLC16A1, CLIC1), tetraspanins such as CD9, CD81, CD82 andtetraspanin-8, and receptors (CD46, CD55, NOTCH1) [23]. Exosomal proteins consists of molecules which are associated with endocytic pathway in cytosol or plasma membrane not in mitochondria, endoplasmic reticulum, and Golgi complex [7].

Besides lipids and proteins, exosomes are also known to contain large amounts of DNA and RNA. EV-DNA, or DNA that is transported within extracellular vesicles [38] ranges from 100 bp to 2.5 kb in size. MicroRNAs (miRNA) and transfer RNAs (tRNAs) are thought to make up 15% of EV-RNA, and RNA repeats making up 50% of EV-RNA [39], based on sequencing of all RNA derived from isolated EVs in serum. Some RNAs, such as those derived from mesenchymal stem cell exosomes, are present in an increased proportion as compared to that present in the origin cells [40]. Although several studies show that RNA can be transferred within exosomes from cell to cell [41, 42], the extent to which this transferred RNA is functional in the recipient cells and the extent of their fragmentation and transfer is yet to be determined.

## Methods of Isolation

Exosomes have shown to be a promising source for potential biomarker, before detecting and quantifying the exosomes, they must be extracted from biological fluids as pure isolates. This section summarizes the various methods currently employed for exosome isolation along with their comparison in Table 3.

**Table 3:** Methods of exosome isolation

Isolation	Principle	Advantages	Disadvantages	References
Ultracentrifugation	Density, size and shape based sequential separations of particulate constituents and solutes. Consists of several centrifugation steps aiming to remove cells and debris, followed by exosomes	Reduced cost and contamination risks Large sample capacity Yields large amounts of exosomes	High equipment cost, long run time, labour intensive, low portability High speed centrifugation can damage exosomes	[43]
Ultrafiltration	Based on size differences between exosomes and other particulates. The exosome population will be concentrated on the pore containing membrane	Fast, does not require special equipment, good portability	Moderate purity of isolated exosomes, shear stress induced deterioration, possibility of clogging and vesicles trapping, exosome loss due to attaching to membranes	[44]
Size exclusion chromatography	Separates macromolecules on the basis of their size, applying fluid on a column packed with porous, polymeric beads	Precise separation of large and small molecules Exosome structure is not affected by shearing force	Long runtime	[45]
Precipitation	Altering the solubility or dispersibility of exosomes by using water-excluding polymers	Easy to use, does not require specialized equipment, large and scalable sample capacity Mild effect on isolated exosomes	Co-precipitation of other non-exosomal contaminants like proteins and polymeric materials Long runtime	[46, 47]
Immunoaffinity-based capture	Exosome fishing based on specific interactions between membrane-bound antigens (receptors) of exosomes and immobilized antibodies (ligands)	Highly purified exosomes High possibility of subtyping	High reagent costs, exosome tags must be known, low capacity and low yields, antigenic epitopes may be blocked or masked Isolated exosomes may lose their functional capacity	[48]
Microfluidic technologies	Manipulates small amounts of fluids using channels with dimensions of micrometres using capillary forces	Small volume sample is required, simple, time-saving and low cost, real time process control	Less sensitive	[49, 50]

### Ultracentrifugation

Ultracentrifugation applies a high centrifugal force of 100,000×*g* to a heterogeneous mixture. Constituents in the mixture are therefore sedimented sequentially according to their density and size, with larger or denser particles sedimenting first. Ultracentrifugation is of two types, the differential centrifugation which involves centrifuging the sample at increasing speeds (300–2000×*g*), to remove large cells and debris and higher speed spin at 10,000×*g* to sediment larger extracellular vesicles and remaining cell debris, and finally, ultracentrifugation at 100,000×*g* to isolate exosomes [51]. However, ultracentrifugation is time-consuming with low exosomal yield and high protein contamination. Sample purity can be increased with the use of a sucrose density gradient, as in density gradient centrifugation. The sucrose density gradient separates particles according to their densities, and exosomes can be found in a 30% sucrose cushion, separated from non-exosomal particles that could otherwise be precipitated during ultracentrifugation. Developing this method further, a one-step sucrose cushion method was demonstrated and has shown to increase the yield in mesenchymal stem cells exosomes isolation [52]. An optimized ultracentrifugation method involving five cycles at 100,000×*g* for 70 min was compared with size exclusion chromatography and found to be removed 95% of serum protein without any significant loss of exosomes [43]. Density gradient ultracentrifugation has shown to isolate human tongue cancer-derived exosomes with an increased extraction efficiency of uniform-sized exosomes by two-folds [53]. Further, an iodixanol density gradient ultracentrifugation method was demonstrated for the isolation of exosome from human saliva. This method isolated exosomes in the size range of 47.8 ± 12.3 nm with higher concentration which was not possible with the commonly used density gradient methods [54]. Isolating exosomes from plasma is more clinically significant as blood plasma can be easily be obtained from patients but contain a higher level of contamination in terms of non EV proteins and lipoproteins regardless of the method for isolation used.

### Ultrafiltration

This size-based isolation method involves the usage of membrane filters with specific size exclusion limits. A 0.22 µm pore size-containing membrane filters can be used to collect exosomes from the filtrate. Before this filtration step, the cells, debris, and larger vesicles must be removed from the exosome-containing fluid by centrifugation steps or by passing through membrane filters of pore sizes such as 0.80 µm from retentate [7, 44, 55]. Exosomes are used to study various diseases along with their underlying medical conditions and for the same, their isolation from body fluids is essential. A centrifugal ultrafiltration approach was demonstrated for isolating exosomes from bronchoalveolar lavage fluid providing information about various respiratory conditions. 100 kDa molecular weight cut-off nano-membrane filter was used in this method and was also compared to the density gradient method for exosome isolation. It was found that the ultrafiltration approach could isolate 7.69 ± 2.6 × 10^8^/μL compared to 0.5 ± 0.05 × 10^8^/μL using the latter as enumerated using NTA. Thus, proving efficient, simple, and high purity exosome isolation [56]. Ultrafiltration has shown to isolate urinary exosomes with much lesser time and more efficiently than ultracentrifugation. A nano-membrane concentrator was used to concentrate the exosomes and showed to detect them in the minimal urine sample volume of 0.5 mL [57]. Further, exosomes from human colon cancer samples were isolated by combining ultrafiltration with sequential centrifugation. The method was able to perform unbiased isolation of exosomes from conditioned media with uniformity in vesicle size [58]. Fetal bovine serum (FBS) is an important supplement for various cell culture related studies and is found to contain a large number of EVs such as exosomes. These vesicles tend to sometime disrupt the experiments by interfering with cell viability and functioning thereby causing bias to the results. An efficient ultrafiltration-based method which used ultracentrifugation combined with commercial Amicon ultra-15 centrifugal filters was demonstrated to complete deplete the FBS of vesicles. This method was found to be more efficient than the commercially available methods with being cost-effective, can be replicated in any laboratory, and supported cell proliferation up to 96 h [59].

### Size-Exclusion Chromatography (SEC)

SEC is another technique that aims to separate molecules based on their size. A mixture is passed down a column of beads containing multiple pores. Individual molecules can pass through the pores in the polymeric beads depending on their size, with molecules with smaller radii being able to pass into the pores and having to migrate through the column’s tunnels and therefore eluting later from the column. Molecules like exosomes, which have larger hydrodynamic radii, are unable to enter the pores, and therefore, pass through the column faster [45].

Exosome isolation is quite challenging as a number of proteins and lipoproteins are present in the plasma membrane. A single step size exclusion chromatography method was demonstrated for isolation of exosomes using a sepharose CL-2B column and found to efficiently separate the vesicles with a diameter of more than 75 nm from body fluids [60]. The SEC method was also found to minimally alter exosome characteristics compared to precipitation-based methods. It was observed that the former method was more efficient in removing the soluble plasma protein and showed the presence of surface markers such as CD-9, CD-63, etc. in the isolated exosomes compared to the precipitation methods [61]. A simple isolation of exosome acute myeloid leukemia (AML) plasma using mini-sized size exclusion chromatography columns packed with sepharose 2B was performed. The method was able to isolate clean and non-aggregated exosomes in the size range of 50–200 nm [62]. Further, the SEC method coupled with ultracentrifugation was found to be more efficient than both the methods stand alone. First using ultracentrifugation, the EV particles were separated from the sample followed by enrichment of exosomes using the size exclusion chromatography. This approach was found to reduce user variability in the experiment along with the improved yield of exosomes [63].

### Precipitation

Precipitation techniques alter the solubility of exosomes in a solvent to precipitate them out of the solution. Generally, water-excluding polymers that attract water molecules to each other and therefore force insoluble molecules in the water out of solution are used for this purpose, particularly polyethylene glycol with 8 kDa molecular weight [46]. Following a short incubation period where the precipitation solution is mixed with the exosome-containing fluid, centrifugation with a tabletop centrifuge can be used to pellet the precipitated exosomes. Commercially available exosome isolation kits also separate exosomes from biological fluids through precipitation, yielding high quantities of protein, miRNA, and mRNA [47].

Apart from these methods, a charged-based precipitation method can also be used for the isolation of exosomes. These EVs are negatively charged which can be easily precipitated by interaction with positive molecules such as Protamine. The exosomes recovered by this method were compared to that isolated using ultracentrifugation and the former showed more efficient recovery when compared by NTA [64]. A low cost and effective method utilizing polyethylene glycol (PEG) as the agent for precipitation was demonstrated. The PEG wraps the exosomes to aggregate them which leads to easy precipitation. The exosomes isolated by this method were compared for the surface marker in the ExoCarta database and found to contain the top 97% of the exosome markers [65]. Further, a rapid and inexpensive method utilizing solvent-based precipitation was used for the isolation of exosomes from human blood plasma. Initially, the plasma proteins were precipitated using cold acetone which left the exosomes in the supernatant. Then either using ultracentrifugation or filtration, the exosomes can be easily separated. This method was found to be efficient in removing plasma protein contamination and took lesser time than traditional ultracentrifugation methods [66].

### Immunoaffinity-Based Capture (IAC)

IAC uses the affinitive binding property of proteins with protein receptors present in the exosome membranes and vice-versa, to specifically isolate exosomes from biological fluids. ELISA is a common method that uses IAC to capture and quantify exosomes, using the exosome biomarkers CD63, CD326, Tim-4 binding phosphatidylserine, etc. [48]. IAC can be used to further purify exosomes isolated using other non-specific techniques, such as those based on density and size. Isolating tumor-specific exosome is a tedious job as they are being very less in number. The IAC-based method was shown to separate and isolate the melanoma-specific exosomes using mAb 763.74 which is specific for CSPG4 epitope expressed by the cells. The efficiency to capture melanoma-specific exosomes was found around 95% with this method thereby proving itself as a method for liquid biopsy [67]. An anti-CD34 antibody-based IAC method demonstrated AML specific exosomes isolation from the supernatant of cell culture supernatant. The CD34 microbeads were found to be highly efficient and 10 µL aliquots of the same was able to capture all the exosomes in 100–1000 µL of AML suspension [68]. Another IAC-based method used anti-epithelial cell adhesion molecule (EpCAM) which were modified by magnetic activated cell sorting (MACS) to isolate ovarian cancer-derived exosomes. The EpCAM molecules tagged to magnetic microbeads were mixed with serum samples and isolated using a MACS separator. The method was able to detect a higher concentration of exosomal protein related to ovarian cancer in patients (0.149 ± 0.065 mg/mL) as compared to normal humans (0.039 ± 0.030 mg/mL). Thus, showing itself as an efficient method to obtain highly pure exosomes [69].

### Microfluidics-Based Technologies

Microfluidics provide highly efficient, rapid methods for the isolation and detection of exosomes on a single chip. Different methods of exosome isolation are employed, all based on size and utilizing nano filters, nanoarrays, or nanowires. The first method includes silicone nanowires engraved on the micropillars’ sidewalls that help to trap liposomes. The second method is acoustic nanofiltration, by which exosomes of size 100–1000 nm are isolated using the microbeads. Another method is viscoelastic microfluidics, in which elastic lift forces determine particle separation of different sizes through a viscoelastic medium such as Poly-(oxyethylene) (PEO). Through this, > 80% isolation efficiency and > 90% purity could be obtained [49]. Isolation based on filtration using ExoTIC includes a microfluidic chip that separates EVs through membranes of different pore sizes [50]. The dialysis membrane (30 nm pore size) is observed to isolate EVs with the application of electric forces [70]. Wu et al. demonstrated a method of isolating exosomes from whole blood using acoustofluidics (a combination of acoustics and microfluidics). It includes a microscale cell-removal module, which removes large blood parts, and the exosome-isolation module, which separates extracellular vesicles sub-parts like microvesicles [71]. Another method is immunoaffinity-based separation integrated with microfluidic devices to isolate circulating extracellular vesicles filled with exosomes from a serum sample of the blood, using ExoChip. This device is made up of polydimethylsiloxane (PDMS), and antibodies were used to functionalize it against CD63 antigen [72]. Isolation of exosomes could be carried out employing one of these techniques, based on the research and analysis requirements. Table 3 summarizes the working principles, advantages, and disadvantages of some common exosome isolation techniques.

## Methods of Quantification

The small size of exosomes makes traditional quantification methods in molecular biology cumbersome and inaccurate. With the advancements in technology and instrumentation, various methods are currently employed for their faster and more efficient quantification. The current methods for quantification, their principles, and their advantages and disadvantages have been discussed herewith along with Table 4 which summarizes all the methods comprehensively.

**Table 4:** Methods of exosome quantification

Method	Principle	Advantages	Disadvantages	References
Nanoparticle tracking analysis (NTA)	Based on the detection of light scatter by particles in suspension and their Brownian motion to estimate the number and volume distribution of EVs	Does not rely on detection of a specific marker Direct quantification	Expensive instrument Photobleaching and potential background from dye aggregates Measures non-exosomal contaminants also	[73, 74]
Flow cytometry	Flow cytometry detects particles suspended in a fluid by their interaction with a laser beam as they flow through a detection cell	Direct quantification	Insensitivity to smaller exosomes. Requires binding to fluorophore-conjugated antibody-coated beads Swarm effect that means multiple smaller vesicles are counted as single particle. This may provide false positive result	[75–77]
Tunable resistive pulse sensing	Detects the passage of individual particles through a pore in a membrane	Direct quantification	Pore clogging. Insensitivity to smaller exosomes. Measures non-exosomal contaminants also	[78, 79]
Electron microscopy	Imaging of individual exosomes under scanning electron microscope	Exosomes are manually counted	Labor intensive, slow process	[80, 81]
Dynamic light scattering	Evaluates fluctuations in the light scattering intensity of particles	High sensitivity, simple sample preparation, rapid	Heterogeneous exosome populations cannot be analyzed, difficulty with polydisperse samples	[82]
Microfluidics-based detection	Transport of fluids controlled by capillary forces	Product purity, high throughput analysis	Not ready for industrialization yet, increase in the signal/noise ratio is encountered	[83, 84]
Surface plasmon resonance (SPR)	A light is focused to a metal film through a prism and the reflected light is detected which is collective oscillation of free electrons. It is sensitive to change in refractive index of the media	Label-free and real-time quantitative analysis technique High sensitivity of up to 1 nM for a 20 kDa protein Specific to the binding event	Difficult to discriminate between specific and non-specific interactions Mass sensitive limitation Limited sensor area Expensive instrument and sensor cost	[85–87]
Single particle interferometric reflectance imaging sensor (SP-IRIS)	A monochromatic light illuminates on sensor surface and scattering signal from individual nanovesicles is detected by *CMOS *camera The signal is enhanced due to the interferometric phenomena	Quantitative, label-free and dynamic detection method Multiplexed phenotyping and digital counting of individual EVs with diameters of 50–200 nm	Detection limit of 3.94E+09 particles/mL	[88–90]

### Nanoparticle Tracking Analysis (NTA)

NTA is used for quantification of exosomes, using the detection of fluctuations of the light scattered by suspended particles due to their Brownian motion to determine the concentration of particles present [91, 92]. A laser beam illuminates to particles suspended in a sample chamber, and a light-sensitive CCD camera mounted on a long working distance microscope acquires a video of the particles in the path of the laser in Brownian motion and scattering light. An external software then analyses the video and tracks individual particles and calculates their hydrodynamic radii with the help of the Stokes–Einstein equation. The particle concentration of the sample can then be determined by counting all particles in the field of view of the camera, giving concentration as the number of particles per cm^3^. NTA is the preferred method for the detection and enumeration of exosomes as it does not detect specific biomarkers and requires no changes to the sample to be made. A fluorescent mode is also available, under which specifically fluorescently-labeled exosomes can be detected, excluding other EVs present in the solution [73]. A drawback to this instrument is that it is limited to the detection of EVs of sizes 30–500 nm and therefore underestimates the concentration of EVs larger than 500 nm [74]. Apart from the use of high cost instrument, thorough knowledge of the software and hardware settings is required to ensure reproducible results. Also, photobleaching as well as potential background from dye aggregates can sometimes interfere with the results obtain.

Gleadle et al. demonstrated the quantification of exosomes in the urine sample from proteinuric patients using NTA. There was a decrease in the particle concentration due to the immunodepletion of albumin in such patients. The particle diameter of 105 nm extracted from the albumin solution by NTA measurements. Hence, the NTA result interpretation requires great caution for the proteins enriched fluids [93]. In the study conducted by Aguilera-Rojas et al. exosome quantification and sizing was performed in blood serum from the dog using this technique. Exosome concentrations in the range of 7.3–17.5 × 10^10^/mL were detected in serum and C2 cell line culture medium [94]. Zheng et al. showed how NTA was used to check the exosome secretion inhibition due to RNA targeted for Rab27a expressed by MDA-MB-231 breast cancer cells. Rab27a and Rab27b were inhibited leading to altered intracellular CD63+ compartments and few exosomes were released into the culture medium. The data obtained proved that NTA was effective in monitoring exosome secretion disruption [95]. NTA is commonly used for EVs measurement. However, similar sizes of lipoproteins could confound the results of EVs quantification. It is suggested that the user strictly follow the protocols and report data accurately [96].

### Flow Cytometry

Flow cytometry is another preferred method for counting exosomes. The instrument can count particles of a size larger than 500 nm and can, therefore, be used to detect microvesicles and apoptotic bodies [75]. However, exosomes lie outside the detection limits of the flow cytometer and must therefore be attached to beads conjugated with specific antibodies against antigens found on the exosome membrane surface. These counting beads are then bound to secondary fluorophore-conjugated antibodies and are suspended in fluid and passed through the center of a detection cell in a thin single particle stream, controlled by a sheath fluid. The fluorophores bound to the beads will fluoresce and emit light of a longer wavelength as an incident laser beam excites them, and the fluorescence detector will measure the fluorescence intensity and the number of emission ‘events’ to count the number of beads, and therefore, exosomes present in the sample [43]. The light scattered by particles will also be detected and measured, with a detector in front of the laser beam detecting forward scatter signals, and a detector at the side of the laser beam detecting side scatter signals. However, this analysis can be disrupted by immune complexes in the sample, which have similar biophysical characteristics as extracellular vesicles and will cause fluctuations in the forward and side scattered light intensities [76]. Another limitation in flow cytometry measurement of exosome is the swarm effect. Multiple smaller vesicles are counted as single event which cause erroneous data interpretation. This occurs if the concentration of smaller vesicles is high in the sample and scattering or fluorescence signal exceed the detection limit [77].

A flow cytometer is the most commonly applied in the analysis of exosomes due to its potentiality to inspect many parameters at the given time. Conventional cytometers could miss the particles with a size below 300 nm due to their side detection limitation. That is why modified new generation flow cytometers having multi-angle lasers could achieve better particle resolution [97]. EVs can also be quantified by on-bead flow cytometry using fluorochrome‐labeled antibodies. The on-bead flow cytometer is standardized for use with the conventional cytometer in the detection and quantification of exosomes [98]. In the study by Rim et al. exosomes consisting of murine lung fibroblasts (Mlg2908) and murine lung cancer cells (LA-4 and KLN 205) were quantitatively measured by fluorescence-activated cell sorting to create a novel tool for observing in vivo genetic alterations during cancer. It was detected that LA-4 lung cancer cells contained increased CD63-specific exosomes. Thereby, helping in the classification of miRNA as diagnostic markers and cancer-specific proteins [99]. To confirm if the microenvironmental acidity is responsible for exosome release and increased prostate specific antigen (PSA) expression during malignancy, nanoscale flow cytometry along with NTA and an immunocapture-based ELISA were performed. This acidity may be an important factor for the detection of cancer, both qualitatively and quantitatively [100]. Again, to establish the experimental reliability of EV under flow cytometry (FC), a working group of experts in EV-FC from ISEV, ISAC and ISTH, developed a consensus framework called MIFlowCyt-EV. Minimum information related to sample preparation, detection, experimental design and analysis should be provided in manuscript on EV-FC results. It does not provide a specific protocol since hardware, methods and software shall continue in evolution in future [101].

### Tunable Resistive Pulse Sensing (TRPS)

TRPS detects individual particles passing through pores in a voltage applied membrane across which voltage is applied [78]. The size of this pore is changeable and can therefore be used for a variety of samples of different sizes. The exosome sample is loaded on one end of the membrane and single exosomes are forced through the pore, decreasing the current flowing through the pore as they pass, due to the increased resistance within the pore at that moment. The fluctuations in the current are detected and analyzed to provide information about the number and size of particles flowing through the pore. A single decrease in the current is called an ‘event’, and the number of events is directly related to the concentration of exosomes in the sample [78]. The disadvantages of this technique include the risk of pores getting clogged with repeated use of the membrane, and the lacking sensitivity of the instrument, whose detection limits do not include small exosomes [79].

Maas et al. showed that the qNano system was used for the determination of the size and concentration of exosomes. The procedure is based on transferring EVs through nanopores and makes it faster and serves small sample volume usage. This real-time calibration technique helps to overcome the challenges faced during the measurement of EVs directly in the fluids [102]. Zhang et al. developed the method to size and quantify the catecholamine molecules in nanometer transmitter vesicles. For this, resistive pulse measurements and vesicle impact electrochemical cytometry (VIEC) as the vesicles leave the nanopore pipet. Bovine adrenal vesicles were analyzed and it showed that vesicles size and counts were varying due to the presence of dense core in the vesicles [103]. Another study conducted by Vogel et al. demonstrated the measurement of EVs concentration from blood plasma using TRPS. Coefficients of the variance of 23.9% and 52.5% were detected for the mean liposome and EVs concentrations, respectively [104]. Bogomolny et al. characterized EVs obtained from bacteria and they are also known as outer membrane vesicles (OMVs) using TRPS. Size distribution (124 ± 3 nm modal diameter) and concentration (lower bound 7.4 × 10^9^/mL) were determined from uropathogenic *Escherichia coli* [105]. Appling TRPS for the analysis of EVs could aid in this particle development in therapeutics and clinical diagnostics.

### Electron Microscopy

Electron microscopy is the most preferred method for attesting the quality of exosome isolation and for ensuring that the vesicles are undamaged. Whole-mount negative staining is a well-characterized and often used technique to image exosomes and display morphology. However, the exosomes are completely spherical, as demonstrated by cryo-TEM imaging, and the cup shape is a result of the drying process for the preparation for imaging. Plastic embedding, blocking sectioning, and fixation with glutaraldehyde can reduce the change in the exosome morphology, and may be preferred to observe the natural structure of extracellular vesicles [80, 81]. While it remains a useful method to confirm the morphology and purity of exosome isolates, electron microscopy is too tedious and low throughput technique for the efficient counting of exosomes. It is also expected to under-represent the number of exosomes present, due to the loss of vesicles during sample preparation for microscopy [106].

Characterization and quantification of the exosomes from the primary culture of the canine transmissible venereal tumor were performed using scanning electron microscopy (SEM) as part of immunotherapy for treating the tumor [107]. EVs from human milk were also quantified using SEM and it was observed that the size of these nanovesicles was in the range between 50 and 350 nm [108]. Transmission electron microscopy (TEM) was employed for the purpose of quantification and characterization of mitochondria-rich cells that provide cellular ions for intestinal homeostasis to study and understand its optimal regulation [109]. A cryo-transmission electron microscope was used to identify and characterize the heterogeneous populations of urinary extracellular vesicles from low centrifugation pellets [110]. Immuno-electron microscopy was utilized for the characterization and quantification of respiratory exosomes and nanovesicles derived from the samples of cystic fibrosis, asthma, and ciliary dyskinesia to understand and analyze their involvement in causing lung damage [111].

### Dynamic Light Scattering (DLS)

DLS is commonly used to determine the size of nanoparticles. In a solution, particles randomly move due to Brownian motion leading to collision among them and resulting in the transfer of energy between them, which results in the movement of the solute particles. The energy transfer majorly effects the smaller particles as they move faster in the solvent than larger particles. An incident light beam directed at the solution will be scattered by particles in the solution in all directions [82]. The fluctuations in the scattered light intensity are detected at a certain angle over time by a fast photon detector and analyzed to provide information about the movement of the particles, which further divulges the particle size and concentration in the solution. Smaller particles, which are moving faster, will cause more fluctuations over time in the scattered light intensity. The fluctuations will be studied by analysing the intensity correlation function (*R*) and the diffusion coefficient (*D*) of the particles. The Stokes–Einstein equation can then be used to relate ‘*D*’ to the radius ‘*R*’ of the particles, thus obtaining the size of the particles present in the solution. DLS will also give a measure of the polydispersity of the solution, with values below 0.1 indicating that the sample contains particles of the same size [112]. Fluctuations in light scattered by moving particles are detected and recorded as a function of time.

DLS has several applications in the quantification of exosomes. For instance, a physical quantification of plasma EVs was carried out using DLS to study and identify these vesicles as potential phenotypic biomarkers of prostate cancer [113]. Microvesicles were prepared from the mesenchymal stem cells using ultracentrifugation to study their biological impact which was evaluated using DLS [114]. Exosomes from human mesenchymal cells were quantified using DLS as the regulation of vascular endothelial growth factors derived from breast cancer tissues were regulated by them [115]. DLS coupled with nanoplasmonic assay improved the quantification of EVs from the samples of multiple sclerosis patients which helped to study the physiological deviation in vesicles [116]. Exosomes incubated from different cell types were loaded with drugs such as doxorubicin and were quantified using DLS to determine the efficacy of the drug packing. It was observed that pancreatic cancer cells were the most efficient drug-loaded exosomes followed by macrophages [117].

### Microfluidics

Microfluidics is a system or a process that is used to manipulate small amounts of fluids, ranging from microliter to milliliter using channels with micro-dimensions. A microfluidic device facilitates immunocapture, quantification and characterization of exosomes in the cell culture medium, as well as a patient sample. Fang et al. used microfluidic devices to detect exosomes that were characterized by TEM. The immune captured exosomes were quantified by a stable on-chip capture efficiency using a programmable pump system [118]. A double-filtration microfluidic device was developed by Liang et al. to isolate and quantify urinary EVs taken from bladder cancer patients based on size-exclusion principle. To quantify EVs, microplates and microchips were developed and a BSA standard solution was used to plot the quantification curve; it was observed that 72.4% efficient compared to ultracentrifugation [83]. Another integrated microfluidic device was developed using RT-PCR for microRNA quantification by Ramshani et al. producing fairly accurate results [84]. Lin et al. discovered the potential for on-chip separation and quantification of exosomes based on deterministic lateral displacement (DLD) chip report. Quantification using this approach can be utilized because of its low sample requirements, low cost, and easy operations [50].

Microfluidics used for exosome quantification has various applications. Sensitive microRNA was detected directly from the derived biological samples using microfluidic exponential rolling circle amplification. The high sensitivity exhibited by this method suggested that the analysis and quantification of miRNA could be done for the application in clinical medicine and biological research [119]. Additionally, to overcome the challenges associated with sensitivity in the quantification of miRNAs, cyclic amplification was coupled with microfluidic Voltage-Assisted Liquid Desorption Electrospray Ionization Mass Spectrometry which suggested that the results were accurate and cost-effective for studying miRNAs in the biomedical samples [120]. Selective quantification of biomarkers in nanovesicles derived from cancerous tissues was performed using microfluidics-based on liquid biopsy screening tests which improved the accuracy of the results [121]. Highly sensitive cancerous exosomes were quantified using a detachable microfluidic device implemented with electrochemical aptasensor (DeMEA) which helped in the early detection of cancerous biomarkers thereby contributing to the early diagnosis (exosome-based cancer diagnosis) [122]. A microfluidic device called ExoChip was designed to isolate and quantify circulating exosomes obtained directly from the serum sample of blood, which are a promising diagnostic biomarkers source and therefore, can be utilized for molecular screening of cancers [72].

### Surface Plasmon Resonance (SPR)

Over the past decade, SPR and its applications have been extensively studied. SPR is a label-free detection technique that employs the resonant oscillation of electrons due to the refractive index mismatch between the surface of the material and incident light [123, 124]. SPR is known to be a reliable platform for studying biomolecule interactions. Furthermore, SPR is also used in the detection and quantification of exosomes. For instance, the concentration of exosomes carrying the tetraspanin membrane protein CD63 was determined using a SPR sensor probed with anti-CD63 antibodies where the SPR response was converted into surface-bound mass. The measurement accuracy was observed to be better than ± 50% [125]. SPR and dual gold nanoparticle (AuNP)-assisted amplification of signal was employed for direct exosome quantification. This method was highly sensitive, differentiating exosomes derived from MCF-10A normal breast cells and MCF-7 breast cancer cells and a 104-fold improvement in the detection limit compared to ELISA was observed. The SPR based sensor also successfully detected the exosomes in 30% fetal bovine serum [126]. SPR integrated antibody microarrays for exosome membrane proteins were used for the real-time quantification of exosomes in tumor cells. A positive correlation was observed between the secretion of exosomes and the metastatic potential of tumor cells [127]. Real-time quantification of clinically relevant exosomes from breast cancer patients was carried out using SPR which could provide information about the stage of a disease stage and enable non-invasive tracking of the tumor-expression levels [128]. Overall, the SPR approach for quantification of exosomes has been a promising technique as it enables real-time detection.

### Single Particle Interferometric Reflectance Imaging Sensor (SP-IRIS)

SP-IRIS is a method for multiplex phenotyping and digitally counting of different populations of exosomes of size greater than 50 nm which are captured using microarray-based solid-phase chips. The IRIS signal depends on the fields interfering after reflecting from the SiO_2_ layer. IRIS exhibits dual-modality consisting of label-free high-throughput measurement of biomass and high-magnification digital detection of single particles [88]. There are many tools used for exosome analysis and characterization, but they may not be very sensitive and the small size of exosomes can cause difficulty. Therefore, a plasmonic microscopy SP-IRIS could analyze the size and antibody interaction of a single exosome with higher sensitivity. Daaboul et al. demonstrated the characterization of exosomes in HEK 293 cell culture from human cerebrospinal fluid (hCSF). The method interferometric imaging could target nanoparticles with size compatibility with exosomes even from a 20 µL of hCSF volume using antibodies against tetraspanins. SP-IRIS could lead to improvements in disease diagnosis [89]. A study by Yang et al. demonstrated label-free imaging and real-time detection of exosomes using SP-IRIS. Exosomes were adsorbed on a chemically modified gold (Au) surface and then intensity of image and size distribution were calculated. The size distribution parameter determines the fusion activity taking place between exosomes and liposomes quantitatively. Antibody-exosome interaction and the behavior of adsorbed exosomes on a surface coated with antibody-coated surface were also monitored [129]. Using defocused images with such interferometric microscopy could improve the detection of nanoparticles such as exosomes and their detectable size limit. Aygun et al. proposed depth scanning correlation (DSC) interferometric microscopy which was able to detect and characterize exosomes over a few nanometers range [90]. Daaboul et al. proposed a label-free microarray imaging method using visible light that captures exosomes using a sensor functionalized by a membrane-specific array capture probe. Exosome populations from pancreatic cancer cell lines were studied by depositing antibodies against CD9, CD63, CD81, Epcam, Tissue Factor, EGFR, Mucin 1, MHC-1, and MHC-2. This technology was used to detect of exosomes from human plasma and it could improve exosome sample preparation standardization allowing liquid biopsy translation based on exosomes [130].

## Functions and Applications

The major property of exosomes in intercellular communication; carrying proteins and genetic information following release from the parent cell into target cells by endocytosis. This function allows these structures to play significant roles in several biological pathways, including inflammation, angiogenesis, coagulation, and apoptosis [131]. Exosomes are also significant in the process of gestation and contribute to the signaling between maternal cells and fetal cells. Exosomes released into the blood circulation from the placenta [132] promote uteroplacental angiogenesis and inhibit the maternal immune response from acting against the growing fetus. They also maintain homeostasis within the cell by secreting out harmful DNA and other cytosolic contents [133]. Stem cell-derived exosomes influence bone and tissue regeneration [134]. Exosomes also reduce organ damage by reducing oxidative stress and ROS accumulation [135]. However, they are implicated in many pathophysiological roles as well, increasing rates of tumor growth, infection, immune suppression, etc. They are involved in the development of cancers, especially in tumor growth, drug resistance, survival, and metastasis. They mediate intercellular communication in hypoxic conditions, which aggravates tumor development; help to establish premetastatic niches required for metastasis; contribute to the tumor microenvironment by transporting cancerous cells to the niche [136, 137]; and prevent the proliferation of lymphocytes, therefore helping tumor cells avoid detection and destruction by the immune system [138, 139]. This role in immune system regulation is highlighted by the mechanism of HIV infection, in which the virus particles are carried into CD4+ cells by exosomes and therefore avoid detection by immune cells [140]. Besides their natural biological functions, exosomes are now being studied for their potential use in diagnostics. Exosomes from the urine samples of renal ischemia, prostate cancer, and reperfusion injury patients contained markers for these diseases that are rarely detected in the whole urine sample. Early detection of lung cancer could become a reality, as exosomes isolated from plasma of lung tumor patients contained lung-tumor associated miRNAs and have higher levels of CAV1 expression than exosomes of healthy people. The concentration of placenta-derived exosomes is higher in expectant mothers with complications such as gestational diabetes [141] and pre-eclampsia [142] than in normal pregnant women. Salivary exosome contents can be used to detect Sjogren’s syndrome and pancreatobiliary tract cancer [143], while cerebrospinal fluidic exosomes contain biomarkers for Parkinson’s disease, Alzheimer’s disease, and such other neurodegenerative disorders such as prion diseases. The molecular contents of exosomes isolated from biological fluids have proven to be a useful tool for the detection of several diseases.

Exosomes are also being investigated for their use as therapeutic agents. Due to their low immunogenicity, low toxicity, biocompatibility, biodistribution, ability to target specific cells and tissues, and ability to transfer their contents into recipient cells via endocytosis, they are ideal for the delivery of therapeutic molecules across biological barriers to target cells. Artificial exosomes containing biological therapeutics, such as siRNA, recombinant proteins, and anti-inflammatory drugs, can be prepared via different methods, such as (1) Exosomes isolation from donor cells, followed by artificial delivery of therapeutics into vesicles; (2) Transfection of therapeutic-encoding DNA into donor cells, which will be expressed and secreted out in exosomes; (3) Coating donor cells with a therapeutic material, which will be drawn into the cell and sorted into exosomes during their biogenesis. The artificial exosomes are then delivered to target cells and unload their molecular cargo into the cells, conferring a therapeutic effect. Alternatively, parent cells can be genetically modified to produce exosomes that contain the required therapeutic molecules [2].

In recent years, major research and progress have been made to use exosomes in diagnostics as most body fluids have exosomes that contain RNA, proteins, and lipids [144]. Eight proteins were identified from urine which constitutes an important diagnostic aspect of bladder cancer [145]. Exosomes are also used in the targeted drug delivery as vehicles, such as the Brain which has a Blood–Brain–Barrier obstacle could also be targeted [146]. Brain inflammation could be treated using drugs such as curcumin encapsulated (Exo-cur) to inhibit the signal transduction and activate transcription thereby enabling the delivery of the drug into the microglia cells [147]. Exosomes are used in immunotherapy, RNA interference as they serve as an excellent therapeutic cargo [148]. As exosomes are involved in cell–cell communication, its role in aging and senescence is notable [149]. Tumor-derived exosomes play an anti-tumorigenic role as they contain tumor-specific antigens [85].

## Conclusion

With the increase of our knowledge about the role of exosomes in diseases, there has been a rapid surge of interest in this field. Depending on the components and quantity of exosomes, researchers can pinpoint their cellular sources and also monitor the progress of the disease. Apart from being a biomarker, exosomes have also shown its role as a therapeutic agent against diseases. A major hindrance in exosome research is the difficulty in isolating pure samples of exosomes and quantifying them directly, due to their small size. Current methods employed for isolation include ultracentrifugation, ultrafiltration, size exclusion chromatography, precipitation, immunoaffinity-based capture. These methods have undergone expeditious development which has made isolation easier and faster with larger and purified yields of exosomes. However, even these methods of isolation pose challenges such as limited efficiency, co-precipitation of non-exosome molecules, and damage to the vesicular structure. Fewer still are the techniques available for determining the exact number of exosomes in a sample. Quantifying exosomes by nanoparticle tracking analysis, flow cytometry, tunable resistive pulse sensing, electron microscopy, dynamic light scattering, surface plasmon resonance and single particle reflectance imaging sensor has allowed direct quantification with high sensitivity and accuracy. However, the instruments employed for these methods are often very expensive and require high maintenance and complicated processing, and they cannot be easily incorporated in resource-limited settings. In the past decade, novel methods such as microfluidic approaches and combinations of existing methods have been tested for their efficiency and accuracy in both isolation and quantification, and are giving optimistic results with high throughput. The development of a rapid, cost-efficient, and simple technique for the exosomes isolation from clinical samples, followed by their accurate quantification, would greatly advance research into their role as disease biomarkers and therapeutic systems.
